# The use of antibiotics in the early stage of acute exacerbation of chronic obstructive pulmonary disease in patients without obvious signs of infection: a multicenter, randomized, parallel-controlled study

**DOI:** 10.3389/fphar.2024.1380939

**Published:** 2024-05-10

**Authors:** Ling Zhou, Yan Deng, Kui Liu, Huiguo Liu, Wei Liu

**Affiliations:** ^1^ Department of Respiratory and Critical Care Medicine, Tongji Hospital, Tongji Medical College, Huazhong University of Science and Technology, Wuhan, Hubei, China; ^2^ Department of Geriatrics, Tongji Hospital, Tongji Medical College, Huazhong University of Science and Technology, Wuhan, China; ^3^ Key Laboratory of Vascular Aging, Ministry of Education, Tongji Hospital, Tongji Medical College, Huazhong University of Science and Technology, Wuhan, China

**Keywords:** antibiotics, acute exacerbation, chronic obstructive pulmonary disease, infection, prognosis

## Abstract

**Introduction:**

Chronic obstructive pulmonary disease (COPD) is a chronic respiratory disease with high prevalence and mortality. In some acute exacerbations of COPD (AECOPD) in patients with no obvious signs of infection, early antibiotic treatment seems to clinically improve the disease, but more studies are needed to determine the prognostic impact of antibiotic treatment in AECOPD patients with no obvious signs of infection.

**Purpose:**

To clarify the impact of antibiotic treatment on the short-term and long-term prognoses of AECOPD patients without obvious signs of infection.

**Methods:**

The impact of the two treatment methods on the prognosis of patients was compared at 30, 90, 180, and 360 days after discharge. A multicenter, randomized, parallel-controlled clinical trial was conducted in a department of respiratory and critical care medicine in Central China. All patients met the inclusion criteria for AECOPD, and the patients were randomly assigned to the antibiotic group or the nonantibiotic group at a 1:1 ratio. Patients in the antibiotic group were given moxifloxacin 400 mg/day intravenously for 7 days. Patients in the nonantibiotic group were intravenously injected with the same amount of normal saline as the amount of moxifloxacin given to those in the antibiotic group for 7 days.

**Results:**

There were 406 patients in the antibiotic group and 410 patients in the nonantibiotic group. During the short-term and long-term follow-ups, the acute exacerbation frequency, intensive care unit (ICU) treatment rate, mortality, and mMRC and CAT scores were not significantly different between the two groups (*p* > 0.05). At the 180- and 360-day follow-ups, the forced expiratory volume in 1 s (FEV1%) and peak expiratory flow (PEF) were not significantly different between the two groups (*p* > 0.05). The 30-day readmission rate was significantly lower in the antibiotic group than in the nonantibiotic group (*p* < 0.05). The time from discharge to the first acute exacerbation was not significantly different between the two groups (*p* > 0.05). The length of the first hospital stay after discharge was significantly lower in the antibiotic group (5.84 days) than in the nonantibiotic group (6.75 days) (*p* < 0.05). At the 30-day follow-up, the acute exacerbation frequency, age, C-reactive protein (CRP) level, and sputum viscosity were significantly greater in the nonantibiotic group than in the antibiotic group (*p* < 0.05). In addition, according to the receiver operating characteristic (ROC) analysis, the frequency of acute exacerbations at the 30-day follow-up was significantly greater in COPD patients aged >62.5 years, with a CRP level >12.56 mg/L or with a sputum viscosity >III, in the nonantibiotic group than in those in the antibiotic group, suggesting that the short-term prognosis was poor.

**Conclusion:**

Patients who are >62.5 years of age, have a CRP concentration >12.56 mg/L, or have a sputum viscosity >III without obvious signs of infection should be treated with antibiotics to improve their short-term prognosis.

**Clinical Trial Registration:**

(https://www.chictr.org.cn), (ChiCTR1800018921)

## Introduction

COPD is a chronic respiratory disease with high prevalence and high mortality that causes considerable health and economic losses worldwide. Globally, more than 3 million patients die from COPD every year, and most of these deaths are caused by AECOPD episodes (Aboumatar et al., 2018). AECOPD is associated with increased airway and systemic inflammation (Dransfield et al., 2019). Effective anti-infect therapy and prevention of acute exacerbations are the major therapeutic objectives for COPD patients.

Bacterial infection in the respiratory tract is the most important cause of AECOPD, and antibiotics are an important strategy for treating AECOPD patients with signs of bacterial infection (Miravitlles and Anzueto, 2013). AECOPD can lead to a significant decline in lung function and affect the quality of life; at the same time, because patients with this disease are prone to recurrent attacks, these patients often need frequent hospitalizations (Price et al., 2014). The common cause of AECOPD is infection (Wang et al., 2020), and 60% of AECOPD patients have definite evidence of infection (Dimopoulos et al., 2012). Other predisposing factors include smoking, surgery, inhaled allergens, air pollution, and pulmonary embolism (Jafarinejad et al., 2017).

Early antibiotic therapy for AECOPD patients with infection can effectively reduce short-term mortality, reduce the treatment failure rate, and improve sputum production (Gupta et al., 2020; Lancaster et al., 2020; Sethi and Aaron, 2020). Wang et al. (2020) reported that there was no difference in the efficacies between antibiotics and placebo in AECOPD patients without infection (Roede et al., 2007), and there is no consensus on the use of antibiotics in AECOPD patients. Due to the limitations, such as the poor accuracy of bacterial smear results, the long time that bacterial culture results take, and the lack of large-scale controlled clinical trials, of the current clinical bacterial examination methods, it is very difficult to determine whether AECOPD patients have bacterial infections (Ditz et al., 2020; D’Anna et al., 2016). In addition, the decision to use antibiotics for treatment in clinical practice largely depends on the experience of clinical workers. Dobler et al. (2020) showed that compared to nonantibiotic treatment, empirical antibiotic treatment for 3–14 days in all AECOPD patients with mild to severe deterioration can increase their symptom relief rate and reduce their treatment failure rate. Vollenweider et al. (2018) found that there was no significant increase in the risk of adverse events in patients treated with empirical antibiotics compared to patients receiving placebo. In terms of the actual diagnosis and treatment, we found that for patients without obvious signs of infection, early empirical use of broad-spectrum fluoroquinolones can help control the disease faster and significantly improve the subjective symptoms of patients. A multicenter, randomized, parallel-controlled clinical trial was conducted to compare the prognosis of AECOPD patients without clear signs of infection to provide new evidence for the use of antibiotics in the diagnosis and treatment of AECOPD patients.

## Materials and methods

### Subjects

A clinical trial was conducted in respiratory and critical care medicine in tertiary hospitals in Central China to explore the use of antibiotics on the prognosis of AECOPD patients without obvious signs of infection and to determine the effects of antibiotics on the prognosis of these patients. Patients admitted from 10/2018 to 12/2019 were categorized into antibiotic and nonantibiotic groups. Using SAS 9.2 statistical software, a random sequence of 816 subjects in the two groups (antibiotic group and nonantibiotic group) was generated at a 1:1 ratio, and the group numbers (001–816) were listed by a random coding table. The researchers assigned each of the subjects to one of the groups in descending order of enrollment. Patients in the nonantibiotic group were intravenously injected with the same volume of normal saline as the volume of moxifloxacin given to those in the antibiotic group for 7 days. To evaluate the therapeutic efficacy of antibiotics on the short-term and long-term prognoses of these patients, 1-year follow-ups were conducted for all patients. The study is registered at the China Clinical Trials Registry (ChiCTR1800018921).

All COPD patients provided informed consent. The inclusion criteria for patients were as follows: 1) aged between 40 and 70 years; 2) had a diagnosis of AECOPD, in which the clinical symptoms included dyspnea, cough and expectoration, sudden changes beyond the daily range of variation that could be defined as AECOPD; and 3) had a forced expiratory volume in 1 s (FEV1%) < 0.7 after the use of a bronchodilator. The exclusion criteria for patients were as follows: 1) had pulmonary infection (any of four signs: fever; recent symptoms of expectoration; white blood cells >10 × 10^9^/L or <4 × 10^9^/L, with or without a neutrophilic left shift; and abnormal chest imaging results); 2) had left ventricular dysfunction [brain natriuretic peptide (BNP) >400 pg/mL]; 3) needed invasive mechanical ventilation; 4) had lower leg edema, right heart failure, or hemodynamic instability; 5) had serious diseases, such as malignant tumors; or 6) had a moxifloxacin allergy.

A subgroup analysis was performed according to CRP, sputum viscosity, FeNO, GOLD, and age. The subgroups included a high-CRP group and a low-CRP group, a high-sputum-viscosity group and a low-sputum-viscosity group, a high-FeNO group and a low-FeNO group, a high-GOLD-grade or a low-GOLD-grade group, and an age≤59 group and an age>59 group.

### Randomization and follow-ups

The study leaders KL and HGL placed the subjects in order of inclusion in either the antibiotic group or the nonantibiotic group. General data, including sputum viscosity, sputum volume, and mMRC score, were collected on the day of enrollment, and clinical symptoms, including sputum viscosity and sputum volume, were assessed during hospitalization. Clinical indicators and mMRC and CAT scores were assessed 1 year after discharge, and the number of acute exacerbations was recorded during follow-up. Pulmonary function, including FEV1% and PEF, was measured at 180 and 360 days of follow-up.

### Endpoints

The frequency of acute exacerbations at 30, 90, 180, and 360 days was the primary endpoint. Mortality was the key secondary endpoint. Other secondary endpoints included the CAT, readmission rate, ICU treatment rate, at 180 and 360 days after discharge.

### Statistical analysis

Data were analyzed using the chi-squared test, Fisher’s exact test, *t*-test, or Mann‒Whitney *U* test. The ROC curve was used to calculate the area under the curve (AUC) and cut-off value. PRISM 8.0 was used for statistical analysis. A two-sided *p* < 0.05 was considered to indicate statistical significance.

## Results

### General data

**FIGURE 1:**
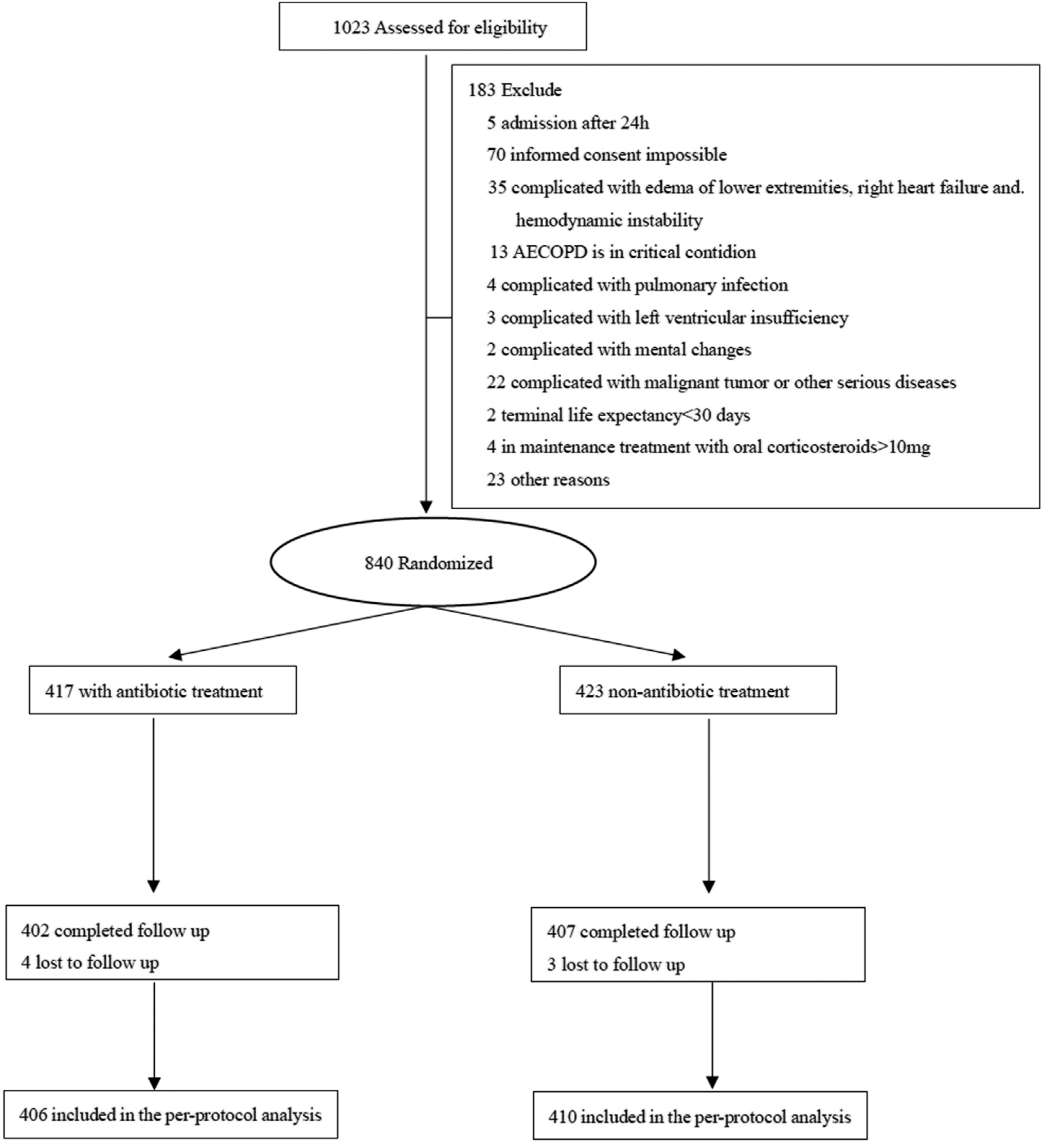
Enrollment, allocation, and follow-up of the trial participants.

The trial participant assignments and follow-ups are shown in Figure 1. In total, 816 COPD patients, including 519 males and 297 females, were included. Age, sex, smoking history, frequency of acute exacerbations in the previous year, admission presentation, pulmonary function, GOLD grade, mMRC score, CAT score, laboratory indicators, and clinical data were not significantly different between the two groups (*p* > 0.05) (Table 1).

**TABLE 1:** General data in antibiotic group and non-antibiotic group.

Items	Total (*n* = 816)	Antibiotic group (*n* = 406)	Non-antibiotic group (*n* = 410)	Statistics	*P*
Age, y	62 (60, 65)	64 (58, 66)	60 (61, 65)	0.610^a^	0.542
Sex					
Men	519 (63.6)	263 (64.8)	256 (62.4)	0.482^b^	0.487
Women	297 (36.4)	143 (35.2)	154 (37.6)		
Smokers				0.915^b^	0.633
Never	470 (57.6)	240 (59.1)	230 (56.1)		
Past	272 (33.3)	132 (32.5)	140 (34.1)		
Current	74 (9.1)	34 (8.4)	40 (9.8)		
Admission presentation					
Cough symptom score	2.05 ± 0.03	2.42 ± 0.03	2.05 ± 0.04	0.169^a^	0.866
Sputum viscosity	1.96 ± 0.03	1.92 ± 0.05	1.99 ± 0.05	1.070^a^	0.285
Sputum volume	1.66 ± 0.02	1.64 ± 0.03	1.69 ± 0.04	1.097^a^	0.273
FEV1%	60 (55, 65)	60 (56, 66)	61 (58, 64)	1.199^a^	0.231
PEF	7.4 (6.4, 8.7)	7.3 (6.6, 8.9)	7.6 (6.4, 8.9)	0.426^a^	0.671
C-reactive protein, mg/L	1.3 (0.02, 3.0)	2.04 (0.63, 3.4)	1.29 (0.15, 3.81)	0.584^a^	0.560
FeNO	18 (9, 23)	19 (15, 25)	10 (12, 21)	1.528^a^	0.127
GOLD				6.083^b^	0.108
GOLD1	60 (7.4)	28 (6.9)	32 (7.8)		
GOLD2	423 (51.8)	198 (48.8)	225 (54.9)		
GOLD3	286 (35.0)	159 (39.1)	127 (31.0)		
GOLD4	47 (5.8)	21 (5.2)	26 (6.3)		
mMRC score	1.61 ± 0.03	1.56 ± 0.04	1.69 ± 0.03	1.914^a^	0.056
CAT score	15.28 ± 0.26	14.87 ± 0.38	15.70 ± 0.35	1.622^a^	0.105

Data were shown as mean ± standard deviation or n (%).

^a^ *t* test.

^b^ χ2 value.

### Endpoints

**FIGURE 2:**
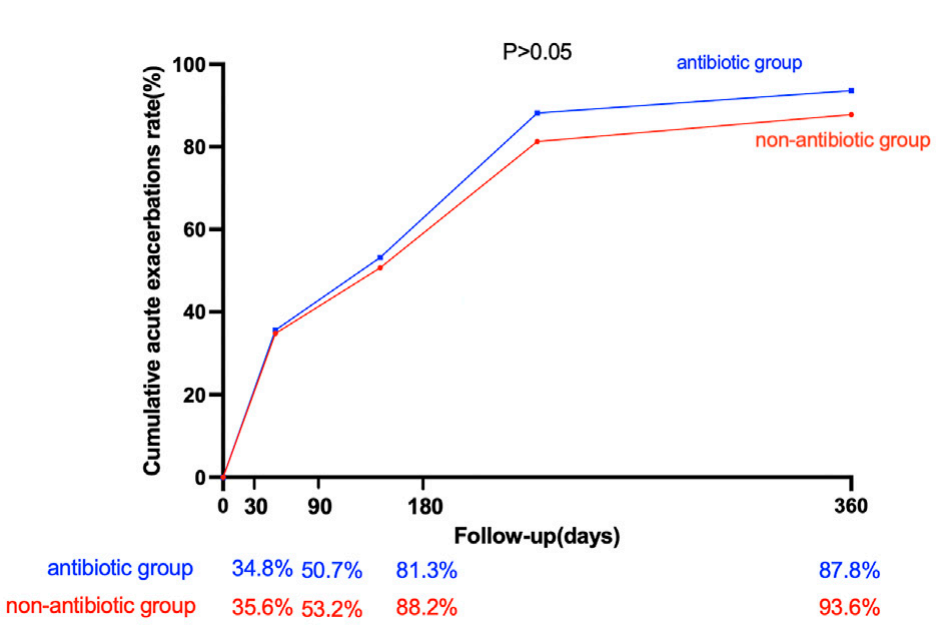
Cumulative acute exacerbation rates in the antibiotic group and nonantibiotic group.

The cumulative acute exacerbation rate in the antibiotic group was greater than that in the nonantibiotic group at 30, 90, 180, and 360 days after discharge, but the difference was not statistically significant (*p* > 0.05) (Figure 2).

The secondary endpoints were mortality, the rehospitalization rate, the CAT score, the ICU treatment rate at 30, 90, 180, and 360 days after discharge. The results showed that the rate of rehospitalization within 30 days was significantly lower in the antibiotic group than in the nonantibiotic group (*p* < 0.05). The time from discharge to the first acute exacerbation in the antibiotic group (45.64 days) was greater than that in the nonantibiotic group (43.97 days), but the difference was not statistically significant between the two groups (*p* > 0.05). The length of hospital stay for the first episode after discharge was significantly lower in the antibiotic group (5.84 days) than in the nonantibiotic group (6.75 days) (*p* < 0.05) (Table 2).

**Table 2:** Secondary end points.

Items	Visit time (Day)	antibiotic group(n = 406)	Non-antibiotic group(n =410)	Statistics	P
Mortality	30	0	0	-	-
	90	2 (0.5)	2 (0.5)	0.009	0.992
	180	4 (1.0)	5 (1.2)	0.103	0.749
	360	7 (1.7)	7 (1.7)	0.003	0.985
CAT score	30	15.83 ± 0.34	15.73 ± 0.36	0.184	0.854
	90	14.57 ± 0.35	15.45 ± 0.31	1.860	0.063
	180	13.86 ± 0.33	14.54 ± 0.34	1.455	0.146
	360	13.85 ± 0.33	14.66 ± 0.30	1.805	0.072
rehospitalization rate	30	75 (18.8)	97 (23.6)	6.505	0.011
	90	116 (28.6)	125 (30.5)	0.360	0.548
	180	201 (49.5)	208 (50.70)	0.122	0.726
	360	275 (67.7)	279 (68.0)	0.009	0.923
ICU treatment rate	30	2 (0.5)	1 (0.2)	0.345	0.623
	90	8 (2.0)	7 (1.7)	0.078	0.780
	180	15 (3.7)	17 (4.1)	0.111	0.740
	360	21 (5.2)	19 (4.6)	0.127	0.722
The time from discharge to first acute exacerbation,day	-	45.64±1.12	43.97±1.57	0.874	0.382
The time of stay in hospital for the first time after discharge, day	-	5.84±0.16	6.75±0.57	1.504	0.026

### Subgroup analysis

#### Subgroup analysis of the outcomes 30 days after discharge

**FIGURE 3:**
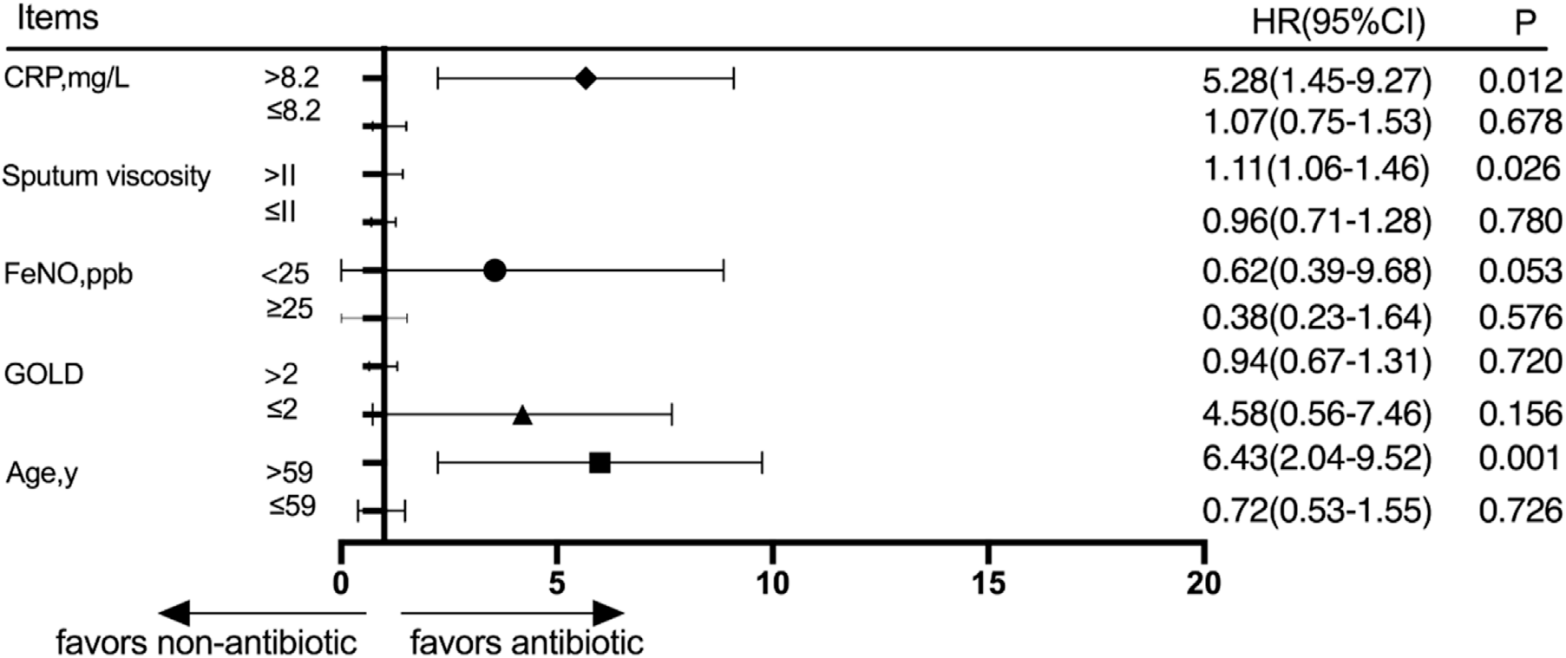
Subgroup analysis was performed according to CRP, sputum viscosity, FeNO, GOLD, and age 30 days after discharge.

Patients were divided into different subgroups according to the CRP score, sputum viscosity, FeNO, GOLD grade, and age. The subjects were divided into a high-CRP group and a low-CRP group according to the CRP value, and there was no significant difference in the cumulative acute exacerbation rate at the 30-day follow-up between the antibiotic group and the nonantibiotic group in the low-CRP group (*p* > 0.05). The patients were divided into a high-sputum-viscosity group and a low-sputum-viscosity group, and the cumulative acute exacerbations rates at the 30-day follow-up were not significantly different between the antibiotic group and nonantibiotic group in the low-sputum-viscosity group (*p* > 0.05). Patients were divided into a high-FeNO group and a low-FeNO group based on whether the patients had a high FeNO or low FeNO level, and the cumulative acute exacerbations rates at the 30-day follow-up were not significantly different between the antibiotic group and nonantibiotic group (*p* > 0.05). The patients were divided into a high-GOLD-grade group and a low-GOLD-grade group based on whether the patients had a high GOLD grade or a low GOLD grade, and the cumulative acute exacerbations rates at the 30-day follow-up were not significantly different between the antibiotic group and nonantibiotic group (*p* > 0.05). The patients were divided into age≤59 and age>59 groups according to age, and the cumulative acute exacerbation rates at the 30-day follow-up were not significantly different between the antibiotic group and nonantibiotic group in the age≤59 group (*p* > 0.05). The results at the 30-day follow-up showed that the cumulative acute exacerbation rate of patients with advanced age, a high CRP score, and high sputum viscosity was significantly lower in the antibiotic group than in the nonantibiotic group, and the difference was statistically significant (*p* < 0.05) (Figure 3).

#### Subgroup analysis of the outcomes at 360 days after discharge

**FIGURE 4:**
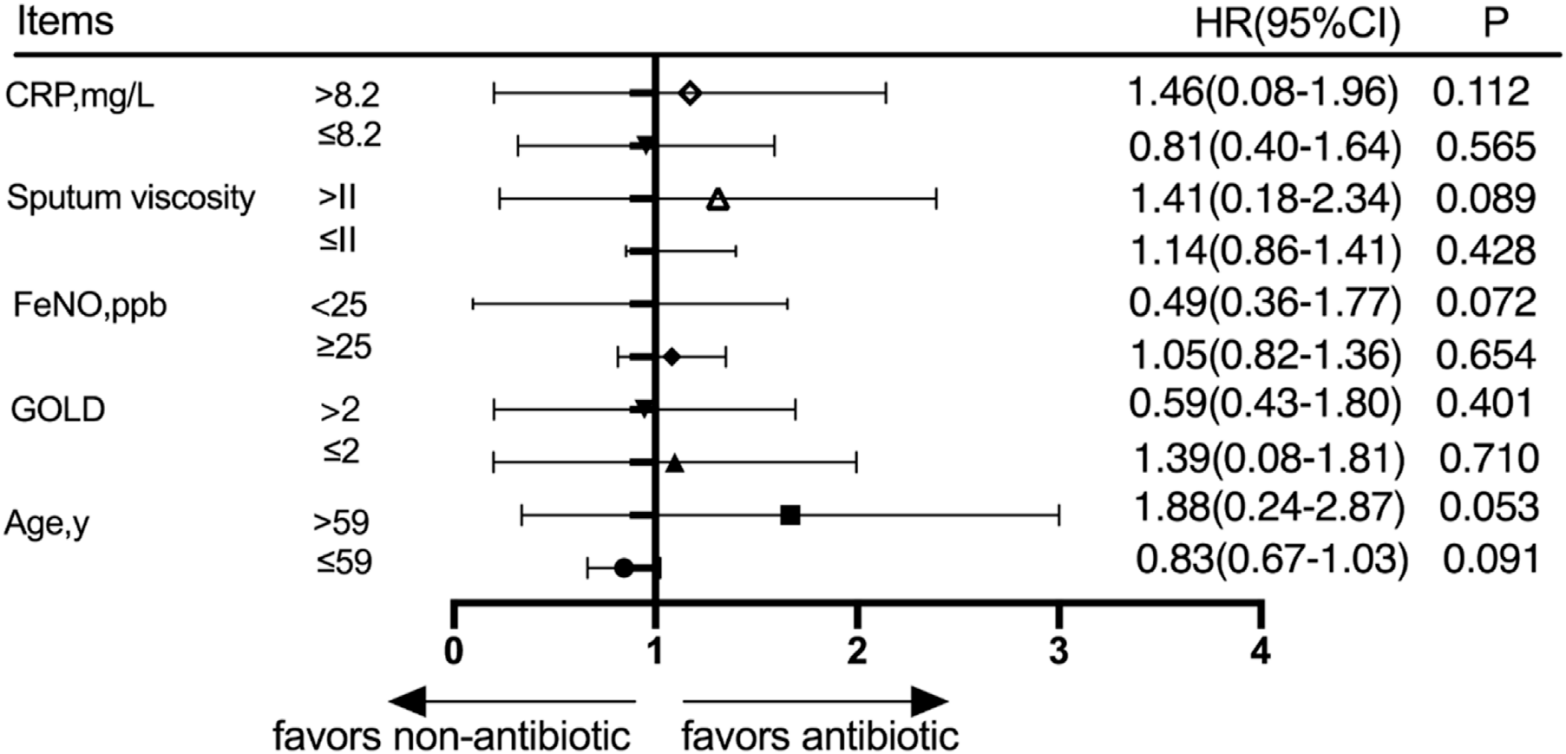
Subgroup analysis was performed according to CRP, sputum viscosity, FeNO, GOLD, and age at 360 days after discharge.

Patients were divided into different subgroups according to the CRP score, sputum viscosity, FeNO, GOLD grade, and age. The subjects were divided into a high-CRP group and a low-CRP group according to the CRP value based on whether the patients had a high CRP or a low CRP, and the cumulative acute exacerbation rates at the 360-day follow-up were not significantly different between the antibiotic group and nonantibiotic group (*p* > 0.05). The subjects were divided into a high-sputum-viscosity group and a low-sputum-viscosity group according to the sputum viscosity based on whether the patient had a high sputum viscosity or a low sputum viscosity, and the cumulative acute exacerbations rates at the 360-day follow-up were not statistically significantly different between the antibiotic group and nonantibiotic group (*p* > 0.05). The patients were divided into a high-FeNO group and a low-FeNO group according to the FeNO value and based on whether the patients had a high FeNO or low FeNO level, and the cumulative acute exacerbations rates at the 360-day follow-up were not significantly different between the antibiotic group and nonantibiotic group (*p* > 0.05). The subjects were divided into a high-GOLD-grade group and a low-GOLD-grade group, and the cumulative acute exacerbation rates at the 360-day follow-up were not significantly different between the antibiotic group and nonantibiotic group in the low-GOLD-grade group (*p* > 0.05). The patients were divided into age≤59 and age>59 groups according to whether the patient was young or elderly, and the cumulative acute exacerbations rates at the 360-day follow-up were not significantly different between the antibiotic group and nonantibiotic group (*p* > 0.05) (Figure 4).

#### Efficacy analysis of each indicator in the evaluation of prognosis

**FIGURE 5:**
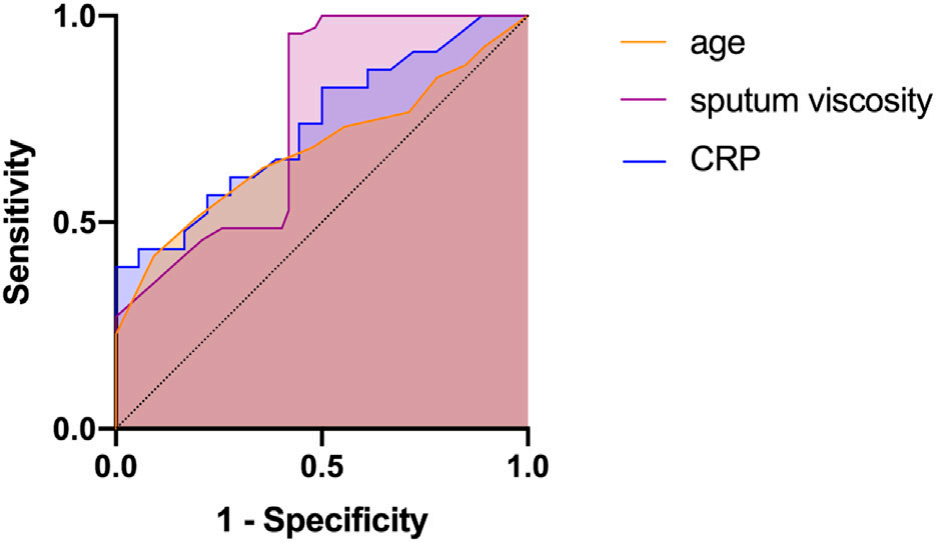
ROC analyses for age, CRP, and sputum viscosity in determining the prognosis.

The subgroup analysis revealed that age, CRP level, and sputum viscosity can affect the results at the 30-day follow-up. ROC curves were used for the significant indices (age, CRP, and sputum viscosity). Age, CRP, and sputum viscosity have diagnostic values for evaluating short-term efficacy (*p* < 0.05). Age >62.5 years, AUC = 0.679 (95% CI: 0.626–0.732); CRP >12.56 mg/L, AUC = 0.733 (95% CI: 0.582–0.884); and sputum viscosity >III, AUC = 0.756 (95% CI: 0.671–0.840) are risk factors for decreased short-term efficacy (Table 3; Figure 5).

**TABLE 3:** Efficacy analysis of each indicators in the evaluation of prognosis.

Items	AUC (95% CI)	*P*	Cut-off	Sensitivity (%)	Specificity (%)	Yoden index
CRP	0.733 (0.582–0.884)	0.011	12.56	42.4%	94.5%	0.369
Sputum viscosity	0.756 (0.671–0.840)	0.001	3	95.7%	59.1%	0.548
Age	0.679 (0.626–0.732)	0.001	62.5	63.9%	52.3%	0.162

## Discussion

The role of antibiotic treatment for patients with COPD without infection is controversial. A reliable indicator of bacterial infection in COPD patients is when sputum has a purulent nature and when there is the presence of Anthonisen type I bacteria during the actual diagnosis and treatment of these patients. We found that in patients without obvious signs of infection, early empirical use of broad-spectrum fluoroquinolones can quickly control the disease and significantly improve the subjective symptoms of these patients. Sanjay et al. showed that with an increase in the sputum volume, exacerbation with dyspnea, and sputum purulence (Jacobs et al., 2019), the absence of prompt antibiotic therapy can be detrimental for many patients with COPD exacerbations (Sethi and Aaron, 2020). Gupta et al. (2020) reported that all COPD patients should be started on empirical antibiotic therapy immediately and that the presence of a *Pseudomonas* infection should mainly be considered. According to the results of an investigative study, the use of antibiotics for AECOPD patients with no obvious signs of infection is significantly greater than that recommended by the guidelines, although the current guidelines do not recommend antibiotic treatment in these patients (López-Campos et al., 2015). To clarify the prognosis of AECOPD patients without clear signs of infection, a multicenter, randomized, parallel-controlled study was conducted, and the results showed that the cumulative acute exacerbation rates at 30, 90, 180, and 360 days after discharge were not significantly different between the antibiotic group and nonantibiotic group.

The CAT is an effective questionnaire for assessing health status (Jones et al., 2009). Yoon et al. (2020) reported that the CAT questionnaire is effective in evaluating the treatment effect of patients with acute exacerbation. Candemir et al. (2015) evaluated the relationship between the CAT score and the improvement of pulmonary rehabilitation (PR) efficiency in COPD patients, and the results showed that the CAT score was moderately correlated with PR efficacy. As an indicator of exacerbations, the mMRC scale is recommended for the assessment of dyspnea and disability (Cheng et al., 2019). The mMRC scale can be used to predict the disease and prognosis, and when the mMRC is ≥ 33, the patient prognosis is significantly worse (Natori et al., 2016). In our study, the mMRC and CAT scores were assessed, and the results showed that there were no significant differences between the antibiotic group and nonantibiotic group on the 30th, 90th, 180th, and 360th days after discharge. Pulmonary function tests can be effectively used to evaluate the lung function of patients. Research has revealed that there is a higher 10-year mortality risk rate in COPD patients when they have a faster annual decrease in their FEV1 (Takei et al., 2019). The PEF value also plays a key role in evaluating the disease status of patients (Cooper et al., 2019). FEV1% and PEF were measured on the 180th and 360th days after discharge, and the results showed that there were no statistically significant differences between the antibiotic group and nonantibiotic group. The results showed that the use of antibiotics had little effect on the prognosis of patients.

In recent decades, clinical doctors and researchers have expanded the treatment goal for COPD because the treatment goal has shifted from improving clinical symptoms to preventing and reducing acute exacerbation (Kim and Aaron, 2018). To obtain a good AECOPD treatment plan, the therapeutic effects on the exacerbation should be considered, and the treatment should also play a role in reducing the number of future acute exacerbations and reducing the severity of future acute exacerbations. These factors have also become an important aspect in evaluating the quality of a treatment plan in clinical practice and research. This study revealed that the readmission rate of patients in the antibiotic group within 30 days after discharge was lower than that of patients in the nonantibiotic group. Studies have shown that readmission within 30 days after discharge in AECOPD patients is related to sex, lung function, systemic glucocorticoid use, Charlson comorbidity index, and quality of medical care (Jo et al., 2020; Yılmaz et al., 2021). Previous studies have shown that antibiotic treatment for AECOPD can significantly prolong the time interval until the next deterioration event (Roede et al., 2008; Roede et al., 2009). Previous studies have also shown that the length of hospital stay can reflect the severity of an AECOPD event (Rinne et al., 2017). The results of this study indicate that antibiotic treatment can reduce the severity of future acute exacerbations in patients with AECOPD without obvious signs of infection.

There are several indicators that are independent prognostic factors in hospitalized patients with AECOPD, but the effect of age on prognosis is still controversial. Crisafulli et al. (2020) showed that age was an independent risk factor for poorer outcomes in patients hospitalized with AECOPD, which is consistent with our findings. The results of the 30-day follow-up showed that the cumulative acute exacerbation rate of patients with advanced age was significantly lower in the antibiotic group than in the nonantibiotic group. The ROC analysis revealed that age >62.5 years was a risk factor for poor prognosis at the 30-day follow-up. Inflammatory factors can not only be used to distinguish viral and bacterial infections in COPD patients but can also predict patient prognosis. Chang et al. (2014) reported that a high CRP level at discharge was an independent predictor of AECOPD readmission. Gao et al. (2017) reported that CRP levels can reflect infection and the level of inflammation and should be used as an auxiliary test for diagnosing AECOPD. Studies have shown that a high CRP level at discharge is an independent predictor of further exacerbation requiring hospital admission for AECOPD, and CRP can serve as an indicator for evaluating the prognosis of AECOPD patients (Chang et al., 2014). Meanwhile, CRP is also considered an effective biological indicator for guiding antibiotic treatment in AECOPD patients, and many studies have advocated for the use of antibiotic treatment for patients with higher levels of CRP (Butler et al., 2019; An et al., 2020). Consistent with this, the subgroup analysis results of this study also showed that for AECOPD patients with high CRP levels, antibiotic treatment can reduce the acute exacerbation rate 30 days after discharge, even if the patient does not have any obvious signs of infection.

Airway mucus hypersecretion refers to the pathophysiological process of excessive mucus production in the airway mucosa and is caused by various pathogenic factors. Under normal circumstances, airway mucus is secreted through the dual control of nerves and the media, and airway mucus can protect the airway and moisten the air. However, under the action of various pathogenic factors, such as smoking and infection, cells in the airway will produce excessive mucus, resulting in cough, expectoration, and even airway obstruction, which will increase the risk of a respiratory tract infection (Lambrecht et al., 2019). In recent years, studies have shown that airway mucus hypersecretion plays an important role in the progression and prognosis of chronic airway inflammatory diseases, including COPD (Shen et al., 2018), and mucus hypersecretion in the airways is an important risk factor for the pathogenesis and progression of chronic airway inflammatory diseases (Xue et al., 2021). Most stable COPD patients have a morning cough with the production of white, foamy sputum. This study revealed that AECOPD patients with no obvious signs of infection who had thick sputum or phlegm and who are undergoing follow-up could have a poor prognosis if they did not receive early treatment with antibiotics. When the disease worsens, the volume and viscosity of mucus in the airway may further increase due to the inflammatory response secondary to infection or other stimuli (Yu et al., 2021). If there is too much mucus in the trachea or if the mucus is too thick, there can be a favorable condition for bacterial colonization in the respiratory tract, causing frequent acute exacerbation and poor prognosis in patients (Miravitlles, 2011). Therefore, the occurrence of viscous sputum or purulent sputum may indicate that the patient has early signs of infection. Antibiotics should be used in these patients as soon as possible to improve their prognosis.

### Limitations

There are still some limitations in our research. Due to the long duration of follow-up and the multicenter nature of the study, patient management was relatively difficult, resulting in a small number of patients being lost to follow-up, and some original data were missing. In this study, the influence of all intervention factors, such as the patient’s geographical region, was not taken into consideration, which may have caused bias in the results. The subgroup analysis of this study was completed after the end of the clinical trial and was exploratory, so the reliability of the conclusions still needs to be verified by further studies.

## Conclusion

In conclusion, this study explored early antibiotic treatment for acute exacerbation of COPD in patients without obvious signs of infection and provides a theoretical basis for individualized treatment of patients with high-risk factors, especially elderly patients, patients with high CRP levels, and patients with a large amount of mucus hypersecretion in their airways. Patients aged >62.5 years, with a CRP level >12.56 mg/L, or a sputum viscosity >III without obvious signs of infection should be treated with antibiotics to improve their short-term prognosis.

## Data Availability

The original contributions presented in the study are included in the article/Supplementary Material, further inquiries can be directed to the corresponding author.
